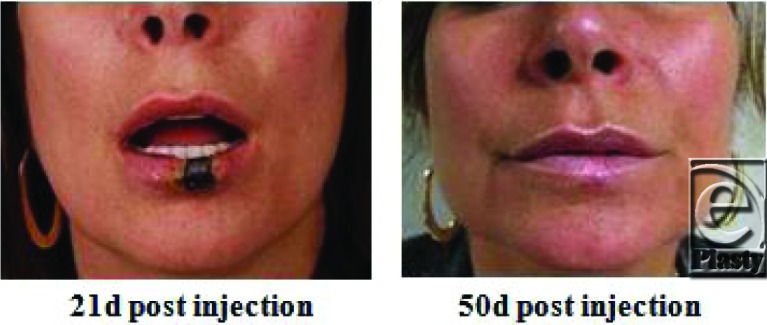# Lower Lip Necrosis

**Published:** 2012-04-12

**Authors:** Alexis Lanteri, Ruth Celestin, Matt Trovato, Gregory Rauscher

**Affiliations:** Department of Surgery, Division of Plastic Surgery, University of Medicine and Dentistry of New Jersey—New Jersey Medical School, Newark, NJ

## DESCRIPTION

A 47-year-old woman presents 12 days after injection of CosmoDerm (Inamed Aesthetic, Santa Barbara, California) to her upper and lower lip by a board-certified plastic surgeon. She reported excruciating pain and blanching of her lower lip at the time of injection and requested the injection be terminated. She had previously undergone multiple CosmoDerm injections in the past 2 years.

## QUESTIONS

1. **Describe the blood supply of the lips.**
2. **Discuss different types of wrinkles (rhytides) and soft tissue filler options.**
3. **Review complications of filler injections.**
4. **Discuss the treatment for lower lip necrosis.**

## DISCUSSION

Soft tissue augmentation dates back more than 100 years and is commonly used for temporary aesthetic enhancement in place of major surgical intervention. A subtle improvement to the lips and surrounding structures by the injection of soft tissue fillers is culturally associated with a more youthful appearance. When performed by an experienced cosmetic physician, filler injection offers high patient satisfaction with low complication rates. However, physicians performing the procedure must be well versed in cutaneous and soft tissue anatomy as structures within the injection field are vulnerable to inadvertent damage.

The superior and inferior labial arteries originate from the facial artery at the angle of the mouth. These vessels course between the mucosa and the orbicularis oris muscle and anastamose at the midline with branches from the contralateral side. The superior labial artery provides blood supply to the upper lip with terminal branches supplying the nasal ala and septum. The inferior labial artery provides blood supply to the lower lip and part of the superior chin.

There are 2 basic types of wrinkles (rhytides), which are classified as dynamic or static. Dynamic rhytides are caused by long-term muscle action and include glabellar, “crow's-feet,” and forehead wrinkles, while static ryhtides are caused by exogenous sources such as smoking and sun exposure. The choice of appropriate cosmetic enhancement depends on the etiology of the wrinkle. Superficial rhytides, most commonly associated with static etiology, are best treated with intradermal injections, while more of the substantial wrinkles, associated with dynamic etiology, require an additional subcutaneous component.

The ideal substance for injection must consistently provide pleasing cosmetic results with low side effects despite repeated use. CosmoDerm (Inamed Aesthetic) is a recombinant human collagen that is grown from a single tissue cell line and is resorbed by 9 months. This temporary soft tissue filler has been used successfully to define the lip roll and is amenable to small rhytid treatment.

There are a variety of complications that may occur with dermal filler injections. A majority of injuries are self-limited and include swelling, redness, discomfort, and bruising at the site of injection. Late adverse reactions such as hypersensitivity, nodule formation, and granuloma have also been reported. A rare, but feared, side effect of soft tissue augmentation is tissue necrosis. During filler placement, interruption of blood flow may occur either through direct intravascular injection of a filler or through compression of vasculature secondary to the increased pressure of the surround soft tissue from the volume of injected material. Impending necrosis is identified by either an immediate local blanching upon injecting a specific region or a delayed form duskiness of the skin after injection. Treatment includes warm compresses and the use of nitroglycerine paste to facilitate vascular dilation and restore blood flow.

Given the wide range of adverse events, it is critical that physicians counsel patients on risks of injectable therapy and educate patients to ensure accurate treatment expectations.

In the discussed case, antibiotics and topical nitro paste 2% were applied every 8 hours to the compromised lower lip tissue to minimize tissue loss. This conservative treatment enabled partial-thickness skin recovery. However, full-thickness injury of the central lower lip persisted and elective excision of the devitalized tissue was performed with wedge resection.

## Figures and Tables

**Figure:**
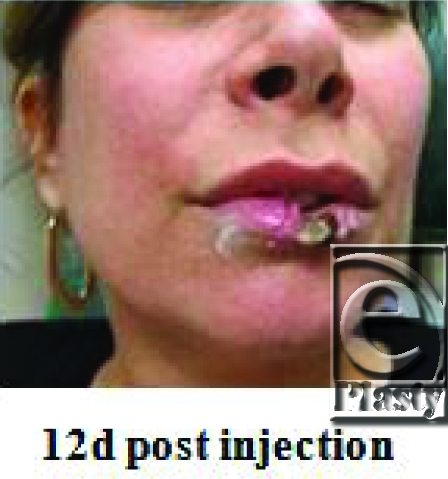


**Figure:**